# Real-life clinical practice with omalizumab in 134 patients with refractory chronic spontaneous urticaria: a single-center experience^☆^

**DOI:** 10.1016/j.abd.2022.06.003

**Published:** 2022-12-23

**Authors:** Elçin Akdaş, Esra Adışen, Murat Orhan Öztaş, Ahmet Burhan Aksakal, Nilsel İlter, Ayla Gülekon

**Affiliations:** Department of Dermatology, Gazi University Faculty of Medicine, Ankara, Turkey

Dear Editor,

Chronic Spontaneous Urticaria (CSU) is associated with high disease burden in terms of health care costs as well as with a reduced performance at work and in social life, and severe deterioration in the quality of life. Second-generation Antihistamines (sgAH) are used in the first steps of CSU treatment because of cheap and safe, but about half of patients are resistant to antihistamines.^1^ Omalizumab, a monoclonal anti-IgE antibody, is an expensive treatment but it is a promising treatment in resistant CSU patients due to its efficacy and safety in long-term use.^2^ The licensed dose of omalizumab in resistant CSU is 300 mg every 4 weeks. The recent international guideline recommends adjusting the dose and interval according to the level of urticaria control.^3^

We evaluated long-term treatment outcomes in a large cohort of patients with resistant CSU who initiated omalizumab 300 mg/4-weeks treatment. Data were obtained from medical records. Patients who received at least 3 doses of omalizumab between October 2014 and January 2021 were included. This study has been approved by the local ethical committee of the Medical University with the Declaration of Helsinki.

In this study, the control of CSU was graded according to the degree of reduction of symptoms reported by the patient and recorded by physician evaluation: ≥ 90% reduction, well-controlled; 30%‒89% reduction, moderately controlled; < 30% reduction, poorly controlled; itch and wheal free, complete remission.

A total of 134 patients were included; 86 (64.2%) female and 48 (35.8%) male aged between 19 to 71 (mean, 42.4 ± 13.1 years). The median duration of urticaria symptoms before omalizumab was 17.5 months (1 to 425 months) (Table 1).

**Table 1:** Baseline characteristics of patients with refractory chronic spontaneous urticaria included in the study.

Patients	Total (n = 134)
Diagnosis, n (%)	
CSU	107 (80)
CSU + CIndU	27 (20)
Age, mean ± SD	42.4 ± 13.1
Sex, n (%)	
Female	86 (64.2)
Male	48 (35.8)
Angioedema, n (%)	71 (53)
Mean age of onset ± SD	37.3 ± 13.4
Duration of urticaria symptoms before OMA, months, median (range)	17.5 (1‒425)
Previous CSU medication other than antihistamine, n (%)	
Systemic corticosteroid	70 (52.2)
Cyclosporine	11 (8.2)
Doxepin	11 (8.2)

CSU, Chronic Spontaneous Urticaria; CIndU, Chronic Inducible Urticaria; SD, Standard Deviation; OMA, Omalizumab.

In total, 113 (84.3%) patients achieved complete remission or well-controlled urticaria symptoms, 17 (12.7%) had moderate-controlled, and 4 (3%) showed poorly controlled symptoms.

The number of treatments continued with omalizumab at a dose of 300 mg/4-weeks were ≤ 6, 7‒15, and ≥ 16 doses for 56/134 (42%), 55/134 (41%), and 23/134 (17%) patients, respectively (Table 2). In total, dose reduction or interval prolongation was tolerated in 53/134 (39.5%) patients, whereas the dose increased in 7/134 (0.05%) patients. It was noteworthy that statistically significant differences were observed between the Body Mass Indexes (BMI) of patients who tolerated a reduction of the dose to 150 mg (24/134), continued with 300 mg (98/134), and increased the dose to 450‒600 mg (7/134) (median, kg/m^2^, 23, 27.4, and 29, respectively) (Kruskal–Wallis test; p = 0.047). In addition, patients who tolerated a decrease to 150 mg had a statistically significantly lower body weight than those who continued with 300 mg and were increased to 450‒600 mg (median, kg, 63, 75, and 75, respectively) (Kruskal–Wallis test; p = 0.040).

**Table 2:** Clinical characteristics of omalizumab in 134 patients with refractory chronic spontaneous urticaria.

The number of doses continued with	First group	Second group	Third group	p-value
OMA 300 mg/4-weeks	≤6 doses	7‒15 doses	≥16 doses	
Patients, n (%)	56 (42)	55 (41)	23 (17)	
Duration of urticaria before OMA, months, median (range)	10.5 (3.2‒43.7)	18 (6‒62)	48 (8‒120)	
Total number of OMA doses, n, median (range)	6 (3‒28)	13 (7‒36)	27 (16‒78)	<0.001^a^
Overall OMA treatment duration, months, median (range)	6.5 (3‒44)	13 (7‒50)	32.5 (16‒72)	<0.001^a^
Doses and intervals change, n (%)				
No change (300 mg/4-weeks)	25 (18.7)	35 (26.1)	14 (10.4)	
Extended intervals (300 mg/5-weeks<)	19 (14.2)	8 (6)	2 (1.5)	
Reduced dose (150 mg/4-weeks)	11 (8.2)	8 (6)	5 (3.7)	
Increased dose (450‒600 mg/4-weeks)	1 (0.7)	4 (3)	2 (1.5)	
Patients continuing on OMA, n (%)	22 (16.4)	30 (22.3)	11 (8.2)	
Patients discontinued OMA, n (%)	34 (22.3)	25 (18.6)	12 (8.9)	
Relapsed disease, n (%)	9 (6.7)	14 (10.4)	5 (3.7)	
Time to relapse, months, median (range)	5 (3.5‒25)	5 (3‒11)	5 (4‒7)	

OMA, Omalizumab.

a p-Value significant at p < 0.05 level.

A total of 71/134 (53%) patients discontinued omalizumab at the end of the follow-up period, 65 of whom achieved well-controlled urticaria or complete remission, and 6 due to unresponsiveness to omalizumab. Patients who discontinued treatment in each of the three groups, regardless of the number of treatments of omalizumab 300 mg/4-weeks re-treated with omalizumab after the same duration (median, 5 months) due to recurrence of symptoms (Kruskal–Wallis test; p = 0.93). Re-treated patients responded to omalizumab similarly to their initial response.

It is interesting to note that the time of suffering from urticaria symptoms before omalizumab was weakly but positively correlated with the number of omalizumab 300 mg/4-weeks achieved by the patients (Spearman rank correlation, *r* = 0.231; p = 0.01). In addition, patients with a history of immunosuppressive therapy received omalizumab 300 mg/4-weeks longer than those without (median 10 vs. 6 doses, Mann–Whitney *U*-test; p *<* 0.001). There was no relationship between concomitant angioedema or inducible urticaria and omalizumab 300 mg/4-weeks treatment duration.

No serious side effects were observed, except for mild side effects that resolved spontaneously within a few days.

This real-life observational study confirmed that omalizumab is a safe and effective agent in patients with CSU resistant to sgAH.^4^, ^5^ The present results revealed that a subset of resistant CSU patients initiated on omalizumab 300 mg/4-weeks achieved well-controlled urticaria symptoms in a short time and the overall duration of treatment was also shorter in those patients, however, in patients receiving omalizumab 300 mg/4-weeks for more than 6 doses, the treatment duration may exceed 1‒3 years. It has been demonstrated that prolonged urticaria symptoms before omalizumab and a history of systemic immunosuppressive therapy prolong the duration of omalizumab 300 mg/4 weeks. Similarly, some authors proposed prolonged CSU symptoms before administration of omalizumab and a history of receiving systemic immunosuppressive therapy are associated with unfavorable treatment outcomes.^6^, ^7^, ^8^ Therefore, adding omalizumab without delay in the early course of CSU resistance to sgAH may shorten the overall duration of omalizumab therapy to reduce the risk of suffering from prolonged increased urticaria activity.

Interestingly, it was noteworthy that despite randomized controlled studies had reported that 300 mg of omalizumab per month was the most effective dose, regardless of the patient's weight,^2^ the present study showed that patients with low body weight and BMI maintained symptomatic control with the 150 mg dose. Similar to the present study’s results, some authors also implicated the use of lower doses, which was usually determined by the patients’ body weight.^9^, ^10^ These findings suggest that BMI may be a valuable predictor for omalizumab dose adjustment.

In addition, the fact that symptoms recurred in a median of 5 months following the discontinuation of treatment, regardless of the number of doses of omalizumab 300 mg/4-weeks, suggests staying at the starting dose for a long time does not contribute to remission.

In this regard, we suggest that once well-controlled symptoms are achieved in CSU patients starting with 300 mg/4-weeks of omalizumab, dose reduction or gradual prolongation of the interval may be attempted. This strategy may provide for early control of spontaneous remission, the possibility of prolonging treatment in patients who need it, as well as cost-effectiveness. Also, symptomatic control in refractory CSU patients with low weight and/or BMI can be achieved with 150 mg, which may facilitate access to omalizumab, especially in underdeveloped or developing countries where the acquisition of the biological is difficult.

## Financial support

None declared.

## Authors’ contributions

Elçin Akdaş: Data collection; analysis and interpretation; critical literature review; preparation and writing of the manuscript; study conception and planning, statistical analysis; approval of the final version of the manuscript.

Esra Adışen: Drafting and editing of the manuscript; approval of the final version of the manuscript; study conception and planning; manuscript critical review.

Murat Orhan Öztaş: Approval of the final version of the manuscript; manuscript critical review.

Ahmet Burhan Aksakal: Manuscript critical review; approval of the final version of the manuscript.

Nilsel İlter: Approval of the final version of the manuscript; manuscript critical review.

Ayla Gülekon: Manuscript critical review; approval of the final version of the manuscript.

## Conflicts of interest

None declared.
